# Intervention Effect of a Soybean-Based Complementary Food Supplement on Anemic Infants in a Poor Rural Region in China: Evidence from Quasi-RCT

**DOI:** 10.3390/children11010013

**Published:** 2023-12-21

**Authors:** Jiyong Yin, Tingting Liu, Jing Sun, Junsheng Huo, Jian Huang

**Affiliations:** 1 Key Laboratory of Trace Element Nutrition of National Health Commission of the People’s Republic of China, Department of Food Science and Technology, National Institute for Nutrition and Health, Chinese Center for Disease Control and Prevention, Beijing 100050, China; yinjy@ninh.chinacdc.cn (J.Y.); huangjian@ninh.chinacdc.cn (J.H.); 2 Key Laboratory of Trace Element Nutrition of National Health Commission of the People’s Republic of China, Department of Central Laboratory, National Institute for Nutrition and Health, Chinese Center for Disease Control and Prevention, Beijing 100050, China; liutt@ninh.chinacdc.cn (T.L.); huojs@ninh.chinacdc.cn (J.H.); 3 Key Laboratory of Trace Element Nutrition of National Health Commission of the People’s Republic of China, National Institute for Nutrition and Health, Chinese Center for Disease Control and Prevention, Beijing 100050, China

**Keywords:** complementary food supplement, multiple micronutrients, supplement, Yingyang Bao (YYB), blood indicator, quasi-randomized controlled trial (quasi-RCT)

## Abstract

The soybean-based Yingyang Bao complementary food supplement represents a special nutritional improvement method for anemic infants in many intervention projects across China, while its benefits lack rigorous evidence. Using a quasi-randomized controlled trial design, which adhered to randomization and control except for the blinding method, 248 anemic infants were divided randomly into an intervention group (128 cases received the Yingyang Bao intervention based on routine feeding) and a control group (120 cases only received routine feeding). Anthropometric indicators and 16 blood indicators were measured at baseline and 1 year after intervention. The levels of hemoglobin, 1,25-dihydroxy vitamin D, homocysteine, retinol, vitamin D_3_, and soluble transferrin receptor and the height–age-Z score and weight–age-Z score of the intervention group were significantly improved after the intervention (*p* < 0.05). The homocysteine level improvement appeared to be moderately negatively correlated with the cobalamin level improvement (*p* < 0.05). The improvements of five indicators were significant correlated with the intervention duration (*p* < 0.05), and the corresponding three significant regression equations could predict the intervention effect and the intervention duration to a certain extent. This quasi-randomized controlled trial provided more convincing evidence that Yingyang Bao can effectively improve three kinds of malnutrition compared to previous research which only adopted self before and after comparison.

## 1. Introduction

Anemia, a condition in which the hemoglobin (Hb) concentration in the blood is lower than normal, has been shown to be a public health problem that particularly affects young children. The World Health Organization (WHO) estimates that 40.0% of all children aged 6–59 months worldwide were affected by anemia, and that there were approximately 269 million children with anemia in 2019 [1]. Anemia affects low-, middle- and high-income countries and has significant adverse health consequences on the growth and development of children, and even on their working abilities in the future, as well as adverse impacts on social and economic development [1]. The WHO states that Africa and South East Asia display the highest effects of anemia on children aged 6–59 months, and the prevalence of anemia in both of these regions was approximately ≥40.0% according to the comprehensive report of WHO in 2023 [1].

Anemia always is also a concerning problem in China. The national prevalence of anemia in children under 2 years (not including those under 6 months) in China displayed a serious value (36.9%) in the most recent investigation in 2020 [2]. Therefore, it has become an important public health issue in China.

In order to effectively prevent, control and reduce anemia and the related nutritional problems linked to anemia, complementary food supplements can play a certain positive effect.

At present, complementary food supplements with multiple micronutrients, which come in three material forms (paste, tablet and sprinkles), are a major improvement solution for anemia in infants all over the world. Yingyang Bao (YYB) is a soybean-based complementary food supplement with multiple micronutrients that was developed in China, containing soybean powder and eight micronutrients including calcium carbonate, ethylenediamine tetraacetic acid monosodium ferric salt (NaFeEDTA), ferrous fumarate, zinc oxide, retinol palmitate, 1,25-dihydroxy vitamin D_3_ (vitamin D_3_), thiamin, riboflavin, folic acid and cobalamin. Therefore, as a fortification means with multiple micronutrients, YYB has been used in various nutrition improvement projects for infants and young children in poor rural regions under the support of various funds, and the aims of these projects were to address basic nutrition problems including anemia among infants and young children, starting in 2012. By 2022, YYB had been administered to 991 poor rural counties in 22 provinces, and more than 13,650,000 infants aged between 6 and 24 months had received a YYB intervention. Before and after these projects, studies were performed on the formulation, manufacturing technology and nutrition effects of YYB [3,4,5,6]. 

The data from both small- and large-population intervention projects have demonstrated YYB’s effects on decreasing anemia prevalence, enhancing cognitive performance and potentially improving stunting, but the trial designs of all of the existing projects only included self before and after comparison observations without a randomized controlled trial (RCT) [7,8,9,10,11,12,13,14]. 

In addition, a total of eight micronutrients are enriched in YYB, but observations on the influence of these nutrients, besides NaFeEDTA and ferrous fumarate, are yet to be cultivated. Therefore, the indicators of the YYB intervention’s effect that have been evaluated in existing research to date only include height, weight and hemoglobin level. Thus, there have not been any studies on the other health benefits of YYB, such as the improvements to immunity or other systems.

Based on the above evaluation statuses for YYB interventions, both domestic and international experts have strongly pointed out [15] that YYB’s nutritional value lacks the rigorous control evidence and the measurements that account for more nutrients, as well as a comprehensive understanding and evaluation of its nutritional benefits, in order to demonstrate YYB’s efficacy.

Thus, whether YYB can effectively improve the anemic status and other health indicators of anemic infants, and whether the current YYB formula is appropriate, as well as whether there are relationships between the duration of YYB intake and the improvement in the level of serum indicators are all topics of interest in this field.

In order to resolve the above questions, to obtain more recognition and to provide more rigorous, true, accurate and sufficient evaluation evidence regarding the YYB intervention’s effects, it is necessary to apply a rigorous trial design involving more evaluation indicators. 

An RCT design can provide the most rigorous and scientific evidence for evaluation research, while it often is not adopted in actual nutritional research. The reason for this is that it is difficult to realize the blinding method in a population experiment of nutritional research, which is necessary for a rigorous RCT design. 

Quasi-RCT is one kind of RCT design which has been used in other medical research [16,17]. Our quasi-RCT design adhered to randomization and control, except for the blinding method. Therefore, its value still is higher than other research works only adopting self before and after comparison. The quasi-RCT design does not fully meet the requirements of RCT design, but it is more suitable for actual nutritional research because we could not use a placebo for the control group after we had confirmed that these infants were anemic. Otherwise, this research would be inhumane. 

Our research attempted to conduct a more rigorous, true, accurate and sufficient evaluation of the intervention effects of YYB with multiple micronutrients on anemic infants aged 6–11 months through a quasi-RCT design with multiple blood indicators, so as to resolve the above questions.

In this research, at baseline and 1 year after intervention, the conducted measurements not only included anthropometric indicators, but also 16 biochemistry indicators from blood samples, which included Hb, serum retinol, 1,25-dihydroxy vitamin D (Vitamin D), Vitamin D_2_, Vitamin D_3_, thiamin (VB_1_), cobalamin (VB_12_), folic acid (FA), homocysteine (HCY), serum ferritin (SF), soluble transferrin receptor (sTfR), hypersensitivity C-reaction protein (hsCRP), retinol binding protein (RBP) and metallothionein (MT), as well as three serum immunology indicators (IgA, IgM and IgG). This efficacy research sought to show the effects of YYB interventions on anemic infants through the comparison of the above data between the control group and the intervention group. Additionally, this research also attempted to establish the relationship between the duration of YYB intake and the improvement in the level of blood indicators through linear regression equation. 

Based on the above design, this quasi-RCT research on the YYB complementary food supplement was conducted in Qingzhen county, a poor rural region in the southwest of China, where the main public health problem among infants in the last two decades has been anemia, as the results of the National Nutrition and Health Surveillance in 2013 show [18]. During 2019 and 2020, 248 infants were successively enrolled in this study, and the intervention time was 1 year (1 bag per day), and the last investigation was performed during 2020 and 2021. The data for the baseline and the last investigations were used to analyze the effects of the YYB intervention as a quasi-RCT principle.

None the previous YYB research adopted such a rigorous, true, accurate and sufficient quasi-RCT design with multiple blood indicators, let alone an RCT design. Compared to these previous YYB research works, it is believed that this research will be able to fill the gap in the research on the rigorous control of YYB’s efficacy, and will obtain more rigorous, true, accurate, sufficient and scientific evidence of the effects of the YYB intervention in improving the anemic status and promoting the overall health of anemic infants. On the other hand, this research will establish a series of comprehensively methodological models for the nutritional evaluation of the YYB intervention’s effect on infants, which has also never been realized any all of the previous YYB research, so as to obtain more direct and basic references regarding how to modify the YYB formulation. 

We hope that this research work will provide rigorous evidence to demonstrate that YYB can comprehensively improve the nutritional statuses of anemic infants aged 6–11 months in China. 

## 2. Materials and Methods

### 2.1. Study Population and Design

This research adopted a quasi-RCT design for infants aged 6–11 months and their caregivers in Qingzhen county, Guizhou province in southwest China. A total of 14 townships and communities in Qingzhen county were enrolled in this research. All of the infants aged 6–11 months between May 2019 and July 2020 in Qingzhen county who underwent the “Physician examination of early development of children” at their local maternal and child health care hospital comprised the background population of this research. Those infants who were preliminarily confirmed as being anemic through this examination were invited to receive further anemic diagnosis, adopting the HemoCue Hb 301 analyzer (HemoCue, Lake Forest, CA, USA) to examine the hemoglobin levels of their fingertip peripheral blood in this research. Then, the confirmed anemic infants, who were confirmed as such according to the cutoff value of hemoglobin (Hb) (110 g/L) according to “the method for anemia screen” [19], were screened out, and they were invited to participate in the further research after their parents or caregivers fully understood the relevant information about informed consent and agreed to undergo the relevant experiment. The caregivers of 248 infants agreed to take part in this study, and these infants were divided into an intervention group (n = 128) and a control group (n = 120) according to the random number table that was composed of the numbers 1 and 2, which was generated automatically by Microsoft Excel 2010 (Microsoft Inc., Redmond, Washington, DC, USA) as a quasi-RCT design. A total of 128 infants assigned the number 1 were in the intervention group and 120 infants assigned the number 2 were in the control group. The 128 infants in the intervention group received a supplement consisting of YYB with multiple micronutrients (1 bag per day) along with routine feeding, while the 120 infants in the control group only received routine feeding for the 1 year after they completed the baseline investigation. The parents or caregivers of all of the anemic infants were informed of the anemic states of their infants before they agreed to participate in the intervention group or in the control group. Then, the professional medical personnel carried out the same education for these parents or caregivers of both groups on how to prevent, control and treat anemia, which mainly included guidance on daily diet and dietary supplements. In this research, the means of routine feeding was a feeding method which involved the daily diet and dietary supplements after the parents or caregivers of both groups fully grasped the information about how to prevent, control and treat anemia. Under the above conditions, it can be considered that the routine feeding of both groups was same, which meant that the control group received the same daily diet and dietary supplements as the intervention group, but the intervention group alone received the YYB intervention in addition to the daily diet and dietary supplements. Due to the limitation of this quasi-RCT design without blinding, subjective bias between the YYB intervention group and the control group may have occurred, due to the fact that the parents or caregivers of the YYB intervention group may have let their guard down for anemia because they excessively trusted YYB after they knew of YYB’s effect, while the parents or caregivers of the control group might have given nutritional supplements to their infants because of their excessive worry. Therefore, the results of this design possibly underestimated the actual effect of the YYB intervention due to the limitation of not using a blinding method. To address this limitation, the professionally medical personnel could reduce its effect through introducing the normal dose and frequency of YYB or other nutritional supplements to the parents or caregivers of both groups in the education program. Finally, the venous blood samples of these infants at baseline and at the last investigation points were collected at Qingzhen Maternal and Children Health Care Hospital, and the detection of blood samples and the data analysis were conducted at the central laboratory of the National Institute for Nutrition and Health, Chinese Center for Disease Control and Prevention (NINH, China CDC). 

### 2.2. Participants and Sample Size 

**Figure 1:**
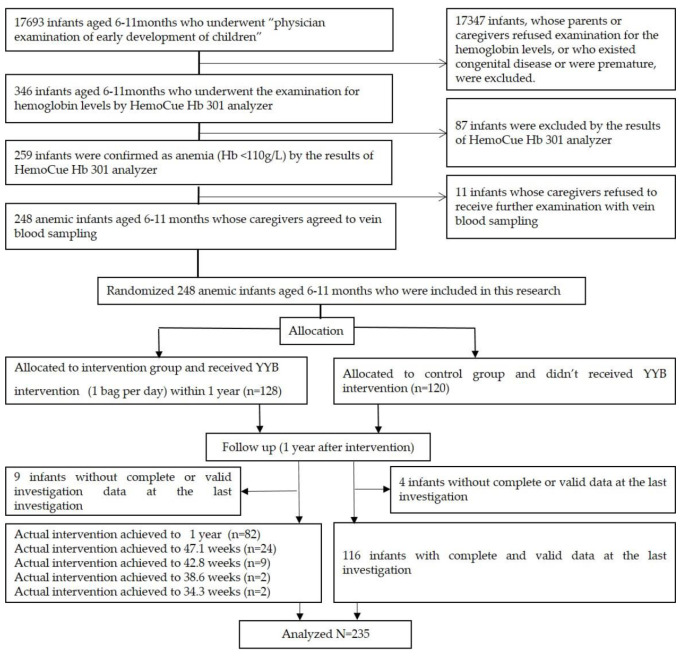
CONSORT flow diagram of this trial in accordance with the statement.

There were total of 17,693 infants aged 6–11 months who were born in Qingzhen county and underwent the “Physician examination of early development of children″ between May 2019 and July 2020. Among these infants, 17,347 infants whose parents or caregivers refused further examination of their hemoglobin levels with the HemoCue Hb 301 analyzer or who displayed congenital diseases or were premature infants were excluded. A total of 259 infants were further confirmed as being anemic by means of the examination of the HemoCue Hb 301 analyzer and according to the cut-off value of Hb (110 g/L) of “the method for anemia screen” [19] after 346 infants underwent examination with the HemoCue Hb 301 analyzer. Among these 259 infants, 11 infants whose parents or caregivers refused further examination with vein blood sampling were also excluded. The remaining 248 infants were divided into an intervention group that received the YYB supplement and routine feeding and a control group that received only routine feeding according to a random number table as per the quasi-RCT design. The duration of the YYB supplement intervention was 1 year, and the intake level of each infant was 1 bag per day. After 1 year, the final data of 235 infants were used to analyze the effect of the YYB supplement because the investigation data of 13 infants (intervention group: 9 infants, control group: 4 infants) that comprised incomplete or invalid information were excluded (Figure 1). Among these 13 infants, the data of 10 infants (intervention group: 8 infants, control group: 2 infants) were missing because we could not contact their parents or caregivers at the last investigation, and the data of 3 infants (intervention group: 1 infant, control group: 2 infants) were outlying data because at least one indicator value exceeded 2SD of the mean. 

The samples were enrolled successively between May 2019 and July 2020, and the overall process of determining the sample size included two stages, which was a process of dynamic determination. At the first stage (from May 2019 to May 2020), the first 11 infants of the intervention group and the first 11 infants of the control group completed the last investigation. Based on the hemoglobin values of the two groups (intervention group: 114.82 ± 5.40 g/L, control group: 112.36 ± 5.48 g/L) at the last investigation point in May 2020, we ultimately determined that the sample size should be 208 anemic infants, including 104 cases in the intervention group and 104 cases in the control group, as an analysis result of the automatic operation of the PASS software (Version 15.0.5). In order to avoid sample loss, we decided to increase the sample size to at least 250 anemic infants according to our previous experience observing a sample loss rate of 16%. However, considering the specific enrollment conditions of the local hospital under the pandemic situation of Coronavirus Disease 2019 (COVID-19) and the overall progress of this research, we had to terminate the enrollment of anemic infants at the end of July 2020. Ultimately, we actually enrolled 248 anemic infants during the whole procedure, meaning that the 248th anemic infant completed the whole procedure between July 2019 and July 2020 and their last investigation was completed at the end of July 2020.

### 2.3. Survey Instrument 

The World Health Organization (WHO) Maternal, Newborn and Child Health Household Survey (WHO, 2009), which included the anthropometric indicators, the feeding information of infants, the two-week prevalence rates of cough and diarrhea of infants pre survey, and the basic personal information of infants and their parents or caregivers, was adopted as a reference framework to implement our survey for the enrolled infants with anemia. 

### 2.4. Training of Implementers 

The professional team was composed of specialists from the NINH, China CDC and the Bill & Melinda Gates Foundation. They provided supervision and training regarding the survey, detection and sample collection. A total of 7 medical personnel belonging to the Qingzhen Municipal Maternal and Child Health Care Hospital were trained on the project for 3 days before this research was implemented (before May 2019). The training contents included the explanation and introduction of the questionnaires involving the basic personal information and dietary information of infants, informed consent, the randomization into groups and the presentation of operations involved in the collection and transportation of blood. After the training, practical operations were carried out within two half days. The assessment requirement of the training was that each medical personnel had to be able to grasp the all of training content so as to be able to conduct the specific operation of all training contents independently. Each problem regarding operation and detection had to be directly analyzed and solved in the training stage. 

### 2.5. The Methods of Collecting Blood Samples and Basic Data

The detection of blood samples involved 4 kinds of equipment, including an automatic biochemical analyzer, an automatic immunoassay analyzer and two liquid mass spectrometry analyzers. Considering the detected serum volume (as least 30 μL for double hole repetition of each sample) of each of the 15 indicators that were detected by the automatic biochemical analyzer, automatic immunoassay analyzer and two liquid mass spectrometry analyzers, the basic sample volume (120 μL) of each analyzer and the volume (at least 500 μL) of the backup sample, it was necessary to collect 1.5 mL serum for each infant. Therefore, 3 mL of venous blood from each enrolled infant was gathered by professional medical personnel at baseline and 1 year after intervention. The venous blood was aliquoted in 3 vacuum freezing tubes (each of the 3 tubes contained 0.5 mL of serum) after it was centrifuged and separated. The sera of the three tubes were used to detect serum retinol, thiamin, cobalamin, folic acid, 1,25-dihydroxy vitamin D_2_ and 1, 25-dihydroxy vitamin D_3_, SF, soluble transferrin receptor (sTfR), hypersensitivity C-reaction protein (hsCRP), retinol binding protein (RBP), homocysteine (HCY), metallothionein (MT) and three immunology indices: IgG, IgA and IgM. The hemoglobin data of these infants came from fingertip blood at baseline and 1 year after intervention. The result of 1,25-dihydroxy vitamin D was the sum of 1,25-dihydroxy vitamin D_2_ and 1,25-dihydroxy vitamin D_3_. At the same time, the data of the basic information and anthropometric measurement of these infants were also collected by one professional medical personnel. 

The “Physician examination of early development of children”, the hemoglobin detection, the anthropometric measurement, and the collection of basic information and blood aliquots were conducted in Qingzhen Municipal Maternal and Child Health Care Hospital at baseline and 1 year after intervention. The tubes with whole blood were left to sit for 20 min, and then were centrifuged at 3000 revolutions per min (RPM) for 15 min. Finally, the sera were separated into the first, the second and the third freezing tubes using disposable pipettes. 

After all blood samples were separated, they were stored at −80 °C immediately, and then they were transported to NINH, China CDC under −80 °C conditions to conduct further serum detection in the laboratory. The correct and strict operations regarding transportation and storage were implemented for avoiding the repeated freezing and thawing of samples.

### 2.6. Laboratory Detection and Analysis 

The height and weight of infants aged 6–11 months were measured using a baby smart physical check-up device at Qingzhen Municipal Maternal and Child Health Care Hospital. The detections of venous blood were implemented in the central laboratory of NINH, China CDC. The SF, sTfR, hCRP, RBP, HCY, MT, IgG, IgA and IgM concentrations were detected by using an automatic biochemical analyzer (Hitachi 7600-210, Tokyo, Japan) according to the requirements of the standard operation of specification, serum folic acid and cobalamin were detected by using an automatic immunoassay analyzer (Roche 601, Basel, Switzerland) according to the requirements of the standard operation of specification, serum 1,25-dihydroxy vitamin D_2_ and 1, 25-dihydroxy vitamin D_3_ were detected by using liquid mass spectrometry (Shimadzu LCMS-8060, Kyoto, Japan) according to the requirements of the standard operation of specification, and serum retinol and thiamin were detected by using liquid mass spectrometry (Waters TQS, Milford, MA, USA) according to the requirements of the standard operation of specification. 

### 2.7. Data Management and Statistical Analysis 

The data for the survey questionnaires were recorded by a special investigator and were inputted into a Microsoft Excel database (Microsoft Inc., Redmond, Washington, DC, USA). The mean and standard deviation (M ± SD) were used to describe continuous variables that were normally distributed, and median and inter-quartile range (M (IQR)) were used to describe variables that were non-normally distributed, and percentages were used to describe discrete variables. The difference in the continuous variable of each indicator between the intervention group and the control group was analyzed using a two-independent-sample *t*-test. For categorical variables including the constituent ratio of gender and the improvement rate, a chi-square test (*χ*^2^ test) and Fisher’s exact test were used to compare the differences between different groups. The height-age-Z (HAZ) score, weight-age-Z (WAZ) score and weight-height-Z (WHZ) score were calculated using the Anthro software (Version 3.2.2, 2011, WHO, Geneve, Switzerland) of the WHO. Correlation analysis was adopted to research the relationships between serum cobalamin and serum HCY, and between the duration of YYB intervention and the improvement in the level of serum indicators. The linear regression equation was constructed as a result of correlation analysis. Statistical significance was set to *p* < 0.05, and all statistical analyses were conducted by using SPSS 16.0 (IBM, Armonk, NY, USA). 

The individual hemoglobin value needed to be calibrated because the altitudes of most of the districts in Qingzhen county are higher than 1000 m, and 110 g/L of hemoglobin was used as the cutoff value for anemia [19] to calculate the prevalence of anemia. As the relevant references, the cutoff value of serum retinol deficiency was 0.1μg/mL [20], that of serum cobalamin deficiency was 196.53 pg/mL according to the recommendation of the reagent kit (Roche, Germany), that of serum folic acid deficiency was 4 ng/mL [21], that of RBP deficiency was 23.1μg/mL [22], that of serum 1,25-dihydroxy vitamin D deficiency was 12 ng/mL [23], that of SF deficiency was 12μg/L [24], that of sTfR was 24.4 nmol/L [25], that of hsCRP was 0.6 mg/L according to the recommendation of reagent kit (Leadman, Shanghai, China), and those of HCY and MT were 15μmol/L and 2509.68 ng/L according to the recommendation of the reagent kit (Leadman, China). In addition, the cutoff values of IgG, IgA and IgM were 6.8 g/L, 0.7 g/L and 0.4 g/L according to the recommendations of the reagent kit (Leadman, China), respectively.

On the other hand, the increase ratio and improvement rate of serum indicators were calculated using Formula 1 and Formula 2, respectively.

Increase ratio = (last measurement value − baseline value)/baseline value(1)

Improvement rate (%) = (the amount of infants who were normal at last investigation while were deficiency at baseline × 100)/the total amount of infants(2)

For missing data, the case would be deleted if the missing information was related to gender, birth date and observation time, or if 1 indicator was missed. In addition, the value of a detected indicator of one sample would be considered as an outlying datum if it exceeded 2 SD of the mean. 

### 2.8. Ethical Approve

This research obtained the authorization of Chinese Clinical Trial Registry, and the ethical approval code was ChiCTR1900028675. 

### 2.9. Participant and Public Involvement 

The participants and the public were not involved in the design and implementation of this research. There are no plans to disseminate the findings of this research to the participants of this research.

## 3. Results

### 3.1. The Basic Characteristics of Anemic Infants 

**Figure 2:**
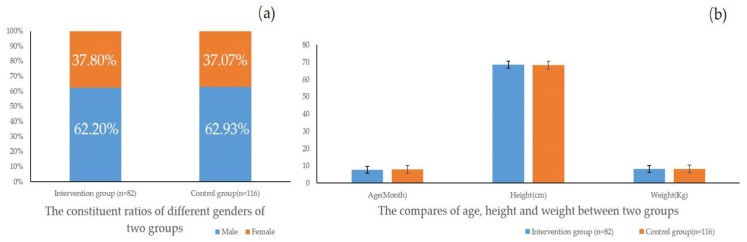
Comparison of the basic characteristics of anemic infants between the two groups before intervention (N = 198). (**a**) The constituent ratios of the different genders of the two groups; (**b**) the differences in age, height and weight between the two group (*p* > 0.05).

The basic characteristics of the intervention group, which included 82 infants who completed the 1 year intervention, and the control group (n = 116) indicated that there were no significant differences in the distribution of the gender, age, height and weight between the intervention group and the control group before the YYB intervention was implemented (Table 1 and Figure 2). For gender, the number of infants of different genders in the intervention group was different from that of the control group, but the differences in the constituent ratios of different genders of the two groups were not significant (*p* = 0.916). For age, the median and inter-quartile range (M (IQR)) were used to present the general condition of each group because this variable had a non-normal distribution. The result indicated that there also was no significant difference in age between the two groups (*p* = 0.317). In addition, the data for both the height and weight of the two groups were normally distributed, and there were also no significant differences between two groups (Height: *p* = 0.825, Weight: *p* = 0.476). Therefore, it was considered that there was comparability between the intervention group and control group (*p* > 0.05).

### 3.2. The Deficient, Insufficiency, and Abnormal Status of 198 Anemic Infants at Baseline 

In the 198 anemic infants, the deficiency rates of VB_1_, FA, VB_12_, iron and RBP were 3.63%, 0.43%, 6.86%, 19.61% and 8.69%, respectively, and the rate of VD insufficiency was 2.26%, while the abnormality rates of sTfR, hsCRP and HCY were 13.28%, 17.52% and 29.50%, respectively, the relatively low rates of IgA, IgM and IgG were 95.33%, 1.91% and 82.49%, respectively, and the stunting rate, low weight rate and wasting rate were 9.27%, 3.74% and 3.74%, respectively, at baseline. The results indicate that deficiencies of VB_12_, iron and RBP, abnormal sTfR and hsCRP, and stunting are mild public health problems, while abnormal HCY and a relatively low IgA and IgG are moderate public health problems in the local region. 

### 3.3. The Comparison of Serum Indicators and Physical Indicators between Two Groups

**Figure 3:**
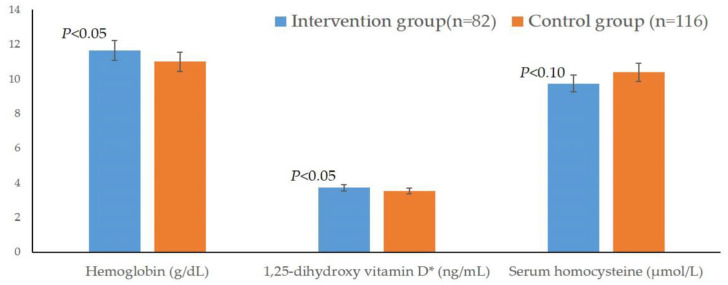
Comparison of Hb, VD and HCY between the two groups after the intervention. *: Data conform to a normal distribution after logarithmic transformation.

The results of the last investigations indicated that the concentrations of Hb and 1,25-dihydroxy vitamin D of the intervention group were significantly higher than those of the control group (*p* < 0.05), and the HCY concentration of the intervention group was significantly better than that of the control group 1 year after the intervention when statistical significance was set at *p* < 0.10 (Figure 3). In addition, the serum retinol, thiamin, 1,25-dihydroxy vitamin D_3_, MT and IgG of intervention group displayed an increasing trend compared to the control group, which meant that these indicators of the intervention group also displayed improvement within 1 year, although the differences between the two groups regarding these indicators were not statistically significant. On the other hand, eight indicators of the intervention group at the last investigation were significantly better than those at the baseline investigation (*p* < 0.05) (Table 2).

### 3.4. The Comparison of the Increased Value Pre and Post Intervention between Two Groups

**Figure 4:**
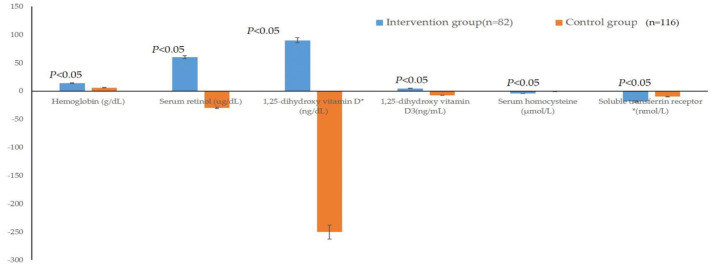
Comparison of the increased values of Hb, Retinol, VD, VD_3_, HCY and sTfR between the two groups after the intervention. *: Data conformity to a normal distribution after logarithmic transformation.

Table 3 indicates the differences in the increased value of each indicator pre- and post-intervention between the two groups. The improvement degrees of Hb, serum retinol, 1,25-dihydroxy vitamin D, 1,25-dihydroxy vitamin D_3_, HCY and sTfR of the intervention group were significantly better than those of the control group (*p* < 0.05) (Figure 4). The serum thiamin, cobalamin, ferritin and IgA also displayed an improvement trend, although the *p* values of these indicators between the two groups were larger than 0.05.

### 3.5. The Comparison of Increase Ratio between Two Groups

**Figure 5:**
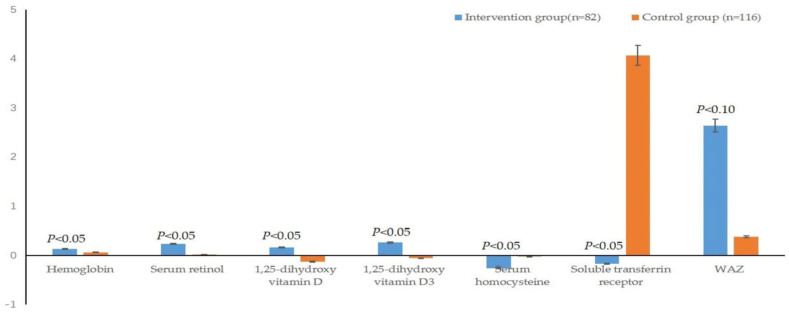
Comparison of the increased ratios of Hb, retinol, VD, VD_3_, HCY and sTfR between the two groups after the intervention.

Table 4 presents the differences in the increase ratios of the 20 indicators between the two groups. The increase ratios of Hb, serum retinol, 1,25-dihydroxy vitamin D, 1,25-dihydroxy vitamin D_3_, HCY and sTfR of the intervention group were significantly better than those of the control group (*p* < 0.05) (Figure 5), and the increase ratio of WAZ of the intervention group was significantly better than that of the control group when the statistically significant level was *p* < 0.10 (Figure 5).

### 3.6. The Comparison of the Improvement Rate between the Two Groups 

**Figure 6:**
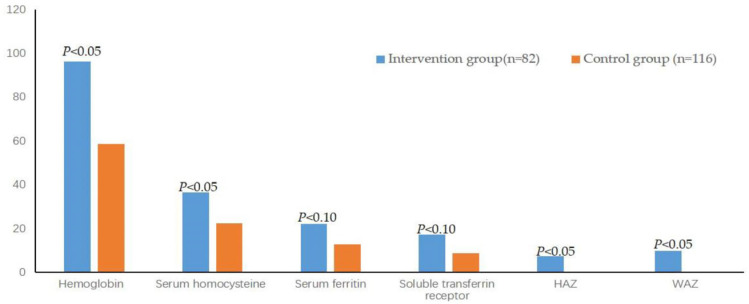
Comparison of the improvement rates of Hb, HCY, SF, sTfR, HAZ and WAZ between the two groups after the intervention.

A total of 12 indicators met the requirements for calculating the improvement rate. Table 5 indicates that the improvement rates of Hb, HCY, HAZ and WAZ of the intervention group were significantly higher than those of the control group (*p* < 0.05) (Figure 6), respectively. The improvement rates of SF and sTfR of the intervention group were significantly better than those of the control group when the statistically significant level was *p* < 0.10 (Figure 6).

### 3.7. The Correlations between the Serum Cobalamin Improvement and the HCY Improvement

**Figure 7:**
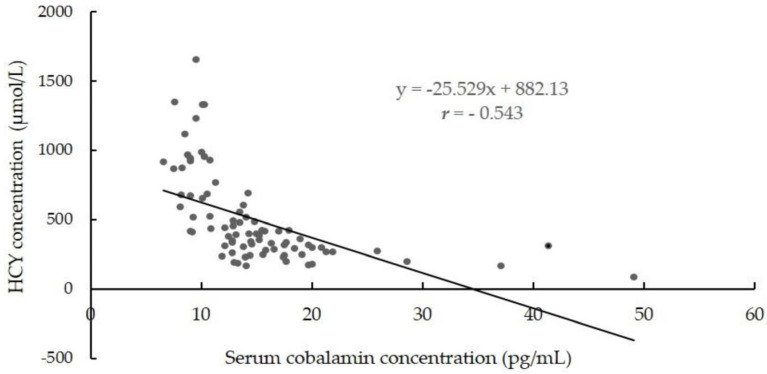
The correlation between serum cobalamin improvement and HCY improvement (n = 82).

The analyses of the correlations between serum cobalamin improvement and HCY improvement and between folic acid improvement and HCY improvement indicated there was a significantly negative correlation between serum cobalamin improvement and HCY improvement in 82 infants who received YYB intervention within 1 year (*r* = −0.543, *p* < 0.05) (Figure 7), while the correlation was not clear between folic acid and HCY. 

### 3.8. The Correlations between the Duration of YYB Intake and the Improvement of Each Indicator

Table 6 indicates that five correlations were significant (*p* < 0.05): the improvement of Hb, cobalamin and IgA displayed strong positive correlations with the duration of YYB intake, and there were strong negative correlations between the duration of YYB intake and improvements in sTfR and HCY (*r* > 0.8, *p* < 0.05), respectively. Another 12 correlations were not significant, although their correlation coefficients indicated a moderate or low correlation, from −0.707 to 0.684 (*p* > 0.05).

### 3.9. Linear Regressions Equations between the Duration of YYB Intake and the Improvement of Each Indicator

**Figure 8:**
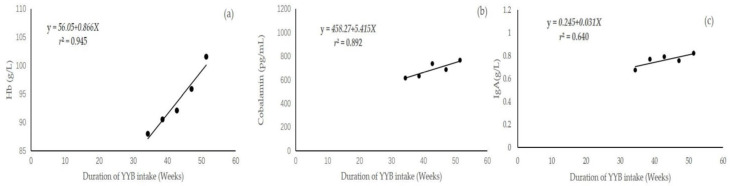
The linear regression equations between the improvement of each of the 3 serum indicators and the duration of YYB intake (n = 119). (**a**) the linear regression equation figure between Hb concentration and the duration of YYB intake; (**b**) the linear regression equation figure between cobalamin concentration and the duration of YYB intake; (**c**) the linear regression equation figure between IgA concentration and the duration of YYB intake.

Table 7 and Figure 8 present the linear regression equations between each indicator (Hb, cobalamin and IgA) and the duration of YYB intake according to the results of the correlations in Table 6. Although the negative correlations between the duration of YYB intake and improvements of sTfR and HCY were also significant, their linear regression equations were not significant. Therefore, the linear regression equations of these indicators were not included. All of the linear regression equations were significant when the statistical significance was set at *p* < 0.10. These equations could be used in predicting the effects of YYB intervention on the improvement of each of the above three blood indicators.

## 4. Discussion 

YYB is a complementary food supplement composed of soybean powder and eight micronutrients, providing a means of fortification with multiple micronutrients that can improve the nutrition state of anemic infants aged 6–11 months in China, and it has been extensively used in rural regions since 2012. In order to better evidence the benefits of YYB with multiple micronutrients, we conducted this rigorous and scientific research with a quasi-RCT design that involved 16 blood indictors in addition to the indicators of anthropometric measurement in Qingzhen county, a poor rural region in the southwest of China. 

This research has greater research value because no team has yet adopted a quasi-RCT design involving 16 blood indictors to explore the nutritional effects of the YYB supplement. Quasi-RCT is one kind of RCT design, and our quasi-RCT design adhered to randomization and control, except for the blinding method. Although our quasi-RCT design is more rigorous, true, accurate and sufficient than previous self before and after comparison observations without RCT, it still has limitations compared to a full RCT, particularly regarding the less robust control of confounding factors, such as the perception of parents or caregivers regarding their infants, which might cause subjective bias. To address this bias, we can reduce its effect through introducing the normal dose and frequency of YYB or other nutritional supplements to the parents or caregivers of both groups in education programs. An RCT design is the most rigorous, true and scientific design, but it is not appropriate in the nutritional field because it cannot meet ethical requirements, because the blinding method in the RCT design would mask the real nutrition state of malnourished infants and only provide a placebo to those in the control group during the whole control process. In our research with quasi-RCT, we informed their parents or caregivers of anemic infants about their actual situation, and conducted education about preventing, controlling and treating anemia to all parents or caregivers, which not only achieved randomization and control but also considered the ethical requirements. Therefore, our quasi-RCT design is not the most rigorous method compared to a full RCT, but it is the most suitable selection in YYB research. In addition, our research adopted four kinds of analytical equipment, which included a HemoCue Hb 301 analyzer, an automatic biochemical analyzer, an automatic immunoassay analyzer and liquid mass spectrometry, to conduct detections for 16 blood indicators. Similar research once validated that the above equipment could be applied to detect the blood indicators of infants [12,26,27,28]. 

On the other hand, no team ever constructed regression equations between the duration of YYB intake and the improvement of nutritional indicators, which can predict the YYB intervention effect under a specific intervention duration or provide a suggestion about the intervention duration to obtain a certain intervention effect. 

Anemic infants aged 6–11 months were selected as the research subjects in this research work. The main reason for this was that anemia is often accompanied by other malnutrition issues and that anemia is a reference indicator of both poor nutrition and poor health, and the results in “Section 3.2” further prove this phenomenon. In 198 anemic infants, deficiencies of VB_12_, iron and RBP, abnormities of sTfR and hsCRP, and the stunting rate were found to be mild public problems, and the abnormity of HCY and a relatively low IgA and IgG were found to be moderate public health problems at the baseline in the local region. In addition, another reason for performing this study was that amenia in infants aged 6–11 months is currently a very serious problem in China [2]. 

This research was conducted only after the parents or caregivers of 248 infants agreed to further examination with vein blood sampling. In this process, the medical personnel of Qingzhen Municipal Maternal and Child Health Care Hospital confirmed that the parent or caregiver of each infant fully understood the relevant information about informed consent and agreed for their child to undergo the further relevant experiment through their signatures on an informed consent form when they enrolled their infants. In addition, infants were excluded from this research if they completely refused vein blood sampling, even if their parent or caregiver had provided informed consent and written their signatures on the informed consent form. 

The results in Table 2, Table 3, Table 4 and Table 5 indicate that YYB can definitely improve Hb, retinol, 1,25-dihydroxy vitamin D_3_, 1,25-dihydroxy vitamin D, HCY, sTfR, HAZ and WAZ at a significance level of 0.05, as well as serum SF and WAZ at a significant level of 0.10. In the field of medical research, some researchers tend to use 0.05 as the standard for testing levels, but 0.10 is also chosen as the testing level in other research works. This approach using a significance level of 0.10 is more risky and is suitable for exploring new methods or intervention measures. Our research aims to explore the actual impact of the YYB intervention on a series of serum indicators including SF and WAZ by using a new research design (a quasi-RCT design). From the biological mechanism, it can be seen that the vitamin D_3_, NaFeEDTA, ferrous fumarate, zinc oxide, retinol palmitate in YYB can promote the changes in serum SF and WAZ. This has a certain biological theoretical basis. In view of this, we conducted this YYB intervention with a quasi-RCT design to explore the actual impact of YYB on serum indicators. In order to better reveal the role of YYB intervention, we would increase the level α to 0.10. Although we increased the probability of Type I errors, we reduced the probability of Type II errors (i.e., in real situations, when factor YYB has an impact on SF and WAZ, but statistical tests cannot detect the probability of YYB’s effect on them), which is more in line with our research goal of reducing false negative conclusions. Indeed, this is indeed an adventurous approach, but it is worth exploring the role of new intervention methods or new evaluation designs. To obtain a better understanding, we further searched for some similar studies [29,30,31], which also used methods with a testing level of 0.10 for research, to provide support for exploring the role of YYB interventions with our new quasi-RCT design. We hoped that the improved effects of SF and WAZ under a significance level of 0.10 would not be covered and that readers would be able to obtain more actual references after they complete their judgement and analysis according to the testing level of 0.10. In addition, this research with a quasi-RCT design provided a new exploration of evaluating YYB’s effect, and our team will conduct further research to fully determine the actually improved effect of YYB for SF and WAZ in the next stage. 

For retinol, cobalamin and 1,25-dihydroxy vitamin D_3_ 1 year after the intervention, as shown in Table 2, the levels of serum retinol and 1,25-dihydroxy vitamin D_3_ in the intervention group were significantly lower than those in the control group at baseline, and their levels in the intervention group displayed a continually increasing trend from baseline to the last investigation point, while the levels of the same two indicators of the control group at the last investigation point were lower than those at baseline. The different trends of the two groups demonstrated that routine feeding in the Qingzhen region could not meet the daily requirements of infants aged 6–11 months for retinol and 1,25-dihydroxy vitamin D_3_, while the YYB supplement is competent in addressing the deficiencies of the two indicators. In addition, the cobalamin level of the intervention group was significantly lower than that of the control group at baseline, while the difference between the two groups was not significant at the last investigation point, which meant that YYB could improve and complement the cobalamin level to a certain extent. The above results were further supported by the increased values and the increase ratios of the six blood indicators, although the increased value and ratio of cobalamin only displayed a trend (Table 3 and Table 4). The above results also prove that YYB can not only improve the anemic status of anemic infants but also can improve other nutritional levels when they are accompanied by other deficiencies. 

As seen in Table 3 and Table 4, the increased values and ratios of Hb, serum retinol, 1,25-dihydroxy vitamin D, 1,25-dihydroxy vitamin D_3_, HCY and sTfR of the intervention group were significantly improved (*p* < 0.05), respectively. In addition, the WAZ of the intervention group was also improved at a significance level of 0.10. These results indicate not only that the anemic statuses of these infants were improved, but also that the possibility of eliminating the potential risks of impairments to vision, growth and development and newborn defects was effectively enhanced by YYB. On the other hand, iron deficiency (ID), which is one of the main reasons for anemia, was controlled to a certain extent by YYB. 

The results in Table 5 show that the improvement effects of YYB on Hb, HCY, HAZ, WAZ, SF and sTfR in the intervention group were significant, which indicated that YYB can relieve the potential risks of newborn defects and growth and development issues in addition to ID in anemic infants aged 6–11 months. Therefore, the health outcomes of this population based on the above three aspects might be better than those without YYB if they are continuously fed with YYB. 

The reason why the results of the improvement rates only included 12 indicators is that some indicators (serum thiamin, 1,25-dihydroxy vitamin D_2_ and 1,25-dihydroxy vitamin D_3_) do not have a cutoff value for judging deficiency, and some indicators (serum retinol, 1,25-dihydroxy vitamin D, cobalamin, folic acid and MT) were not deficient at baseline. 

Table 5 also indicates that the iron deficiency (ID) degree might be more serious than that of folic acid and cobalamin deficiencies in these infants, which meant that ID may be the main reason for the anemia of these infants in the region.

The results in Figure 2 indicate that the supplementation of cobalamin in YYB might directly improve the HCY levels of infants aged 6–11 months. This result is the same in similar studies [32]. The reason for this is that the supplemented cobalamin can promote the function of methionine synthase, which enhances methionine synthesis. Then, the process of HCY being converted into methionine will be improved, and the HCY concentration will decrease after that. The reason why the correlation was not clear between the folic acid improvement and HCY improvement might be that the 82 infants had deficiencies in both cobalamin and folic acid, and the folic acid improvement can only display an obvious relationship with the HCY improvement when the cobalamin concentration is appropriate [33,34]. Additionally, the possible correlations among other indicators need more support from more research. 

The results in Table 6 and Table 7 could be used to predict the duration of YYB intake and the improvement effect of YYB when an improvement in the three indicators needs be realized for the infants in the southwest region of China. For example, the improved Hb value can be roughly estimated using the regression equation of Hb improvement and the duration of YYB intake (Y = 56.05 + 0.866X) after an anemic infant ingests YYB, which would be approximately increased to 99.35 g/L after they continuously ingest YYB for 50 weeks, if their Hb value is 56.01 g/L before they ingest YYB. On the other hand, we can use the regression equation to predict the duration of YYB intake in order for the Hb value of an anemic infant to recover to a normal level. For instance, when an infant’s Hb value approximately is 56.01 g/L before they ingest YYB, they need to continuously ingest YYB for 63 weeks according to the above regression equation if their Hb value is to recover to a normal level (110 g/L). At the same time, these data could be used to provide references for accurate and scientific adjustments to the YYB formulation, drawing up new intervention schemes and proposing new intervention plans when the improved effect needs to be more obvious or the duration of YYB intake needs to be shortened. 

Although this study did not indicate that YYB could significantly improve the nutritional status of other indicators except for Hb, VD, HCY, iron status and so on in the results in Section 3.2, some indicators (such as serum retinol, thiamin, 1,25-dihydroxy vitamin D_3_, MT and IgG) still displayed an improved trend at the last investigation point. The results show that the corresponding micronutrient contents of the current YYB formulation might not be sufficient or accurate for the above indicators, which means that the current YYB formulation should be appropriately and accurately adjusted and modified according to the actual nutritional requirements of infants in the local region of future applications of large-scale YYB interventions in China. Therefore, we should construct a dynamic feedback mechanism between the YYB’s production enterprises and nutrition improvement programs involving a larger population of infants and young children from different regions, and the YYB’s formulation including eight nutrients, provided by production enterprises, should be adjusted and modified depending on the results of the baseline investigation of small-scale populations before the programs involving a larger population are implemented. It can be considered that this mechanism might promote scalability and sustainability among all YYB improvement programs, because it would enable more malnourished infants to obtain more nutritional benefits from YYB. Certainly, in order to verify whether YYB can improve these indicators after adjusting and modifying the formulation, we also need to conduct further research with quasi-RTC for these indicators. On the other hand, the intervention effects of the above indicators might also be significantly improved when the intervention duration is extended. 

We hope that our research can promote broader public health strategies for combating anemia and malnutrition in rural China through these more rigorous, accurate and scientific data about YYB’s efficacy. On the one hand, we might use these data to submit a research report for administrative departments in charge of health, which would potentially promote the wider application of YYB intervention for anemia and malnutrition in rural China, and may also help to reform the strategy of YYB interventions if adopted. On the other hand, these data might provide references for other research works or projects related to YYB interventions for improving anemia and malnutrition in various regions, which would potentially accelerate the adjustment to YYB formulations. Both of the above two aspects would enable YYB to play a more targeted and precise role in improving the nutritional status of malnourished infants in various rural regions of China. 

This research has proved that current YYB interventions can improve the anemic status and other health indicators of anemic infants, and the current formula needs appropriate adjustment regarding the contents of five other nutrients, including NaFeEDTA, vitamin D_3_ and retinol palmitate, when we want to improve the health of more anemic infants in Qingzhen county, China. In addition, we proved that there are correlations between the duration of YYB intake and the improvement effects of five serum indicators. The above findings not only show YYB’s effects on these improved serum indicators, but also indicate the potential for YYB improving other serum indicators if we appropriately adjust the current formulation of YYB. At the same time, the above findings further provide support and references for the theory of nutrition improvement. On the other hand, this evidence will accumulate valuable scientific data for assessing the benefits of YYB as an approach to nutrition promotion among infants and young children. Based on the above analysis, further research should be conducted to explore YYB formulations based on different nutritional requirements, and to investigate the regularities between YYB interventions with different formulations and health outcomes, which will contribute to determining which YYB formulation is optimal formulation for anemic infants with different nutrition problems in different rural regions of China. 

Although this research has obtained valuable evidence about YYB’s efficacy compared to previous YYB researches, we must acknowledge that this research might obtain better results and more valuable evidence if the number of anemic infants in the intervention group could be maintained at 128 cases, and if the actual intervention duration of all anemic infants in the intervention group was 1 year. In addition, we must point out that there may be some biases in this study, including the type of food diversification for the children evaluated, the level of family social characteristics and the knowledge of the parents regarding health–diet–anemia risk between the two groups in this research work. The control group could be used to overcome the above possible biases to a certain extent, but we still hope to achieve better control of these biases through more a rigorous design in future research. Finally, establishing how to overcome or avoid the interference of important events, such as the COVID-19 pandemic, should be a noteworthy issue in future research to a certain extent. 

Therefore, another study about YYB interventions was also conducted by our team in the central region of China. We hope to further and again prove the above results of this research through this new research work, and to obtain more information about the YYB intervention’s effect, so as to find more benefits of the YYB supplement for children, the correlations among different nutrients, and more dose–response relationships between YYB intake and other health statuses through a multicenter clinical trial composed of two similar experiments with a quasi-RCT design on the basis of the two research works in the next stage. 

## 5. Conclusions

The YYB supplement, an important means of fortification with multiple micronutrients, can effectively improve the anemic status of anemic infants, as well as the hyper homocysteine level, iron deficiency (ID) level, growth and development, and immunity when anemic infants simultaneously display the above nutritional deficiencies in the poor rural region in the southwest of China, which means that YYB not only resolves the anemic problem of these infants but also improves four other public health problems that accompany anemia. The results also indicate that the reason why abnormal HCY is improved is derived from the cobalamin supplementation through YYB. A total of three linear regression equations between the duration of YYB intake and the improvements of three blood indicators were constructed. The above findings can be used as references to design more rigorous, scientific, accurate and reasonable YYB formulations, or to draw up better YYB intervention schemes in the future. Finally, the current YYB formulation should be appropriately and accurately adjusted and modified according to the actual nutritional requirements of different regions of China, which will be a future research direction of our research team. 

## Figures and Tables

**Table 1:** The differences in the basic characteristics of anemic infants between the two groups before intervention (N = 198).

	Gender		Age (Month, Median, Inter-Quartile Range)	Height (cm, Mean ± SD)	Weight (Kg, Mean ± SD)
	Male % (n)	Female % (n)			
Intervention group (n = 82)	62.20 (51)	37.80 (31)	7.70 (2.10)	68.42 ± 2.70	8.21 ± 1.08
Control group (n = 116)	62.93 (73)	37.07 (43)	7.85 (2.40)	68.31 ± 3.40	8.10 ± 1.01
*p* value	0.916		0.317	0.825	0.476

**Table 2:** Comparison of serum indicators and physical indicators (M ± SD, M (IQR)).

	Baseline Investigation		Last Investigatoin	
	Intervention Group (n = 82)	Control Group (n = 116)	Intervention Group (n = 82)	Control Group (n = 116)
Hemoglobin (g/L)	103.00 (4.00)	105.00 (6.00)	116.50 (9.00) ^ab^	110.00 (7.00) ^b^
Serum retinol (ug/mL)	0.35 ± 0.10 ^a^	0.40 ± 0.14	0.40 ± 0.11 ^b^	0.37 ± 0.11 ^b^
1,25-dihydroxy vitamin D * (ng/mL)	3.62 ± 0.24 ^a^	3.82 ± 0.34	3.72 ± 0.31 ^a^	3.53 ± 0.33 ^b^
1,25-dihydroxy vitamin D_2_(ng/mL)	1.13 (4.26)	0.74 (5.03)	0.88 (2.10)	0.60 (1.19)
1,25-dihydroxy vitamin D_3_(ng/mL)	34.49 ± 11.42 ^a^	43.32 ± 16.48	39.14 ± 14.22	35.81 ± 12.63
Serum thiamin (ng/mL)	3.85 (3.97)	3.77 (7.48)	4.02 (2.53)	3.68 (2.96)
Serum cobalamin (pg/mL)	6.04 ± 0.59 ^c^	6.20 ± 0.64	6.54 ± 0.49 ^b^	6.56 ± 0.50 ^b^
Serum folic acid (ng/mL)	18.49 (4.13)	18.13 (5.60)	13.28 (9.43)^b^	15.81 (7.07)^b^
Serum homocysteine (μmol/L)	14.92 ± 6.95 ^a^	12.77 ± 7.08	9.73 ± 2.32 ^bc^	10.39 ± 2.60 ^b^
Serum ferritin (ng/mL)	31.50 (34.00)	42.00 (38.50)	39.50 (25.00)	46.50 (33.50)
Soluble transferrin receptor (nmol/L)	0.00 (0.00)	0.00 (0.00)	0.00 (0.00)	0.00 (0.00)
Hypersensitivity C-reaction protein(mg/L)	0.00 (0.26)	0.00 (0.14)	0.00 (0.10)	0.00 (0.11)
Serum retinol binding protein (mg/L)	35.65 (9.92)	32.65 (9.95)	36.70 (8.40)	37.25 (10.25) ^b^
Serum metallothionein (ng/L)	3883.81 ± 324.65	3723.17 ± 332.52	3944.38 ± 358.05	3836.11 ± 324.96
IgA * (g/L)	−1.65 ± 0.77	−1.66 ± 0.91	−0.59 ± 0.80 ^b^	−0.79 ± 1.00 ^b^
IgM * (g/L)	−0.19 ± 0.37	−0.17 ± 0.44	0.16 ± 0.34 ^b^	0.22 ± 0.44 ^b^
IgG * (g/L)	1.62 ± 0.34	1.55 ± 0.38	1.97 ± 0.31 ^b^	1.93 ± 0.44 ^b^
HAZ	−0.38 ± 1.05	−0.56 ± 0.99	−0.55 ± 0.96	−0.75 ± 0.96
WAZ	−0.11 (1.37)	−0.21(1.18)	−0.38 ± 1.03	−0.40 ± 0.91
WHZ	0.29 ± 0.96	0.20 ± 0.98	−0.05 (1.03)	−0.07 (1.01)

*: Data conform to a normal distribution after logarithmic transformation. ^a^: Compared with control group, *p* < 0.05; ^b^: compared with baseline, *p* < 0.05; ^c^: compared with control group, *p* < 0.10.

**Table 3:** Comparison of the difference of increased value of each indicator (M ± SD, M (IQR)).

	Intervention Group (n = 82)	Control Group (n = 116)	*p* Value
Hemoglobin (g/dL)	14.00 (13.00) ^a^	6.00 (10.00)	<0.001
Serum retinol (ug/mL)	0.06 ± 0.14 ^a^	−0.03 ± 0.18	<0.001
1,25-dihydroxy vitamin D * (ng/mL)	0.09 ± 0.38 ^a^	−0.25 ± 0.46	<0.001
1,25-dihydroxy vitamin D_2_ (ng/mL)	−0.20 (2.23)	−0.17 (3.56)	0.463
1,25-dihydroxy vitamin D_3_ (ng/mL)	4.65 ± 16.33 ^a^	−7.52 ± 20.45	<0.001
Serum thiamin (ng/mL)	0.70 (4.50)	−0.11 (6.29)	0.169
Serum cobalamin (pg/mL)	272.62 ± 502.43	187.93 ± 491.66	0.238
Serum folic acid (ng/mL)	−4.09 ± 5.63 ^a^	−1.57 ± 5.96	0.003
Serum homocysteine (μmol/L)	−4.15 (6.60) ^a^	−0.60 (7.10)	<0.001
Serum ferritin (ng/mL)	9.00 (48.00)	7.50 (48.00)	0.914
Soluble transferrin receptor * (nmol/L)	−18.66 ± 43.93 ^a^	−9.97 ± 52.37	0.049
Hypersensitivity C-reaction protein (mg/L)	0.06 (0.25)	0.06 (0.17)	0.874
Serum retinol binding protein (mg/L)	−0.01 (0.46)	0.112 (0.46)	0.061
Serum metallothionein (ng/L)	60.57 ± 491.21	112.94 ± 456.69	0.442
IgA * (g/L)	1.07 ± 1.17	0.87 ± 1.51	0.287
IgM * (g/L)	0.36 ± 0.49	0.38 ± 0.68	0.817
IgG * (g/L)	0.35 (0.73)	0.50 (0.80)	0.640
HAZ	−0.20 (0.92)	−0.21 (0.85)	0.929
WAZ	−0.29 (0.87)	−0.08 (0.95)	0.056
WHZ	−0.25 (1.17)	−0.40 (0.74)	0.323

*: Data conformity to a normal distribution after logarithmic transformation; ^a^: compared with control group, *p* < 0.05.

**Table 4:** Comparison of the increase ratio of each indicator between the two groups (M ± SD, M (IQR)).

	Intervention Group (n = 82)	Control Group (n = 116)	*p* Value
Hemoglobin	0.13 (0.13) ^a^	0.06 (0.10)	0.000
Serum retinol	0.23 ± 0.44 ^a^	0.02 ± 0.46	0.002
1,25-dihydroxy vitamin D	0.16 ± 0.44 ^a^	−0.13 ± 0.38	0.000
1,25-dihydroxy vitamin D_2_	3.23 ± 11.51	3.87 ± 12.30	0.710
1,25-dihydroxy vitamin D_3_	0.26 ± 0.73 ^a^	−0.06 ± 0.46	0.001
Serum thiamin	0.35 ± 1.03	0.66 ± 3.00	0.317
Serum cobalamin	1.14 ± 1.65	0.89 ± 1.40	0.258
Serum folic acid	−0.21 ± 0.33 ^a^	0.03 ± 0.62	0.001
Serum homocysteine	−0.26 ± 0.28 ^a^	−0.03 ± 0.44	0.000
Serum ferritin	3.87 ± 10.33	3.46 ± 12.17	0.809
Soluble transferrin receptor	−0.17 ± 0.38 ^a^	4.07 ± 20.68	0.030
Hypersensitivity C-reaction protein	2.99 ± 19.35	23.62 ± 181.18	0.228
Serum retinol binding protein	0.11 ± 0.46	1.15 ± 5.83	0.059
Serum metallothionein	0.02 ± 0.12	0.04 ± 0.13	0.270
IgA	5.13 ± 11.18	4.70 ± 11.01	0.790
IgM	0.61 ± 0.82	0.83 ± 1.34	0.159
IgG	0.58 ± 0.77	0.76 ± 0.94	0.166
HAZ	0.52 (0.42)	0.375 (0.45)	0.356
WAZ	2.64 (0.37) ^b^	0.38 (0.55)	0.084
WHZ	0.86 (0.87)	2.00 (0.24)	0.162

^a^: Compared with control group, *p* < 0.05; ^b^: compared with control group, *p* < 0.10.

**Table 5:** Comparison of the improvement rate between the two groups (n, %).

	Improvement or Non-Improvement	Intervention Group (n = 82)	Control Group (n = 116)	*p* Value
Hemoglobin	Improvement	79 (96.3) ^a^	68 (58.6)	0.000
	Non-improvement	3 (3.6)	48 (41.4)	
Serum homocysteine	Improvement	30 (36.6) ^a^	26 (22.4)	0.029
	Non-improvement	52 (63.4)	90 (77.6)	
Serum ferritin	Improvement	18 (22.0) ^b^	15 (12.9)	0.093
	Non-improvement	64 (78.0)	101 (87.1)	
Soluble transferrin receptor	Improvement	14 (17.1) ^b^	10 (8.6)	0.073
	Non-improvement	68 (82.9)	106 (91.4)	
Hypersensitivity C-reaction protein	Improvement	13 (15.9)	14 (12.1)	0.445
	Non-improvement	69 (84.1)	102 (87.9)	
Serum retinol binding protein	Improvement	5 (6.1)	13 (11.2)	0.218
	Non-improvement	77 (93.9)	103 (88.8)	
IgA	Improvement	33 (40.2)	45 (38.8)	0.938
	Non-improvement	49 (59.8)	71 (61.2)	
IgM	Improvement	1 (1.2)	3 (2.6)	0.501
	Non-improvement	81 (98.8)	113 (97.4)	
IgG	Improvement	40 (48.8)	57 (49.1)	0.960
	Non-improvement	42 (51.2)	59 (50.9)	
HAZ	Improvement	6 (7.3) ^a^	0 (0.0)	0.005
	Non-improvement	76 (92.7)	116 (100.0)	
WAZ	Improvement	8 (9.8) ^a^	0 (0.0)	0.000
	Non-improvement	74 (90.2)	116 (100.0)	
WHZ	Improvement	7 (8.5)	13 (11.2)	0.539
	Non-improvement	75 (91.5)	103 (88.8)	

^a^: Compared with control group, *p* < 0.05; ^b^: compared with control group, *p* < 0.10.

**Table 6:** The correlations between the duration of YYB intake and the improvement of each blood indicator in the intervention group (n = 119).

	*r*	*p* Value	Correlation Degree
Hemoglobin	0.972	0.028	Strong positive correlation
Serum retinol	0.356	0.488	Non-correlation
1,25-dihydroxy vitamin D	0.343	0.506	Non-correlation
1,25-dihydroxy vitamin D_2_	−0.448	0.373	Non-correlation
1,25-dihydroxy vitamin D_3_	0.684	0.134	Non-correlation
Serum cobalamin	0.944	0.005	Strong positive correlation
Serum thiamin	0.032	0.952	Non-correlation
Serum folic acid	−0.707	0.181	Non-correlation
Serum homocysteine	−0.844	0.034	Strong negative correlation
Serum ferritin	0.327	0.527	Non-correlation
Soluble transferrin receptor	−0.945	0.004	Strong negative correlation
Hypersensitivity C-reaction protein	−0.080	0.881	Non-correlation
Serum retinol binding protein	0.495	0.318	Non-correlation
Serum metallothionein	−0.019	0.971	Non-correlation
IgA	0.800	0.056	Strong positive correlation
IgM	−0.353	0.493	Non-correlation
IgG	0.621	0.188	Non-correlation

**Table 7:** The linear regression equations between the improvement of each of the 3 serum indicators and the duration of YYB intake (n = 119).

	Linear Regression Equation	*p* Value	*r* ^2^
Hemoglobin	Y = 56.05 + 0.866X	0.028	0.945
Serum cobalamin	Y = 458.27 + 5.415X	0.005	0.892
IgA	Y = 0.245 + 0.031X	0.086	0.640

## Data Availability

The data are not publicly available due to their containing information that could compromise the privacy of research participants.
